# Economic impact of prolonged tracheal extubation times on operating room time overall and for subgroups of surgeons: a historical cohort study

**DOI:** 10.1186/s12871-024-02862-6

**Published:** 2025-01-04

**Authors:** Franklin Dexter, Anil A. Marian, Richard H. Epstein

**Affiliations:** 1 Department of Anesthesia, Division of Management Consulting, University of Iowa, 200 Hawkins Drive, 6-JCP, Iowa, IA 52242 USA; 2 Department of Anesthesiology, Perioperative Medicine and Pain Management, 1611 NW 12, University of Miami, Miami, FL 33136 USA

**Keywords:** Airway extubation, Operating rooms, Models, Statistical, Anesthetics, Inhalational

## Abstract

**Background:**

Prolonged tracheal extubation time is defined as an interval ≥ 15 min from the end of surgery to extubation. An earlier study showed that prolonged extubations had a mean 12.4 min longer time from the end of surgery to operating room (OR) exit. Prolonged extubations usually (57%) were observed during OR days with > 8 h of cases and turnovers, such that longer OR times from prolonged extubation can be treated as a variable cost (i.e., each added minute incurs an expense). The current study addressed limitations of the generalizability of these earlier investigations.

**Methods:**

The retrospective cohort study included cases performed at a university hospital October 2011 through June 2023 with general anesthesia, tracheal intubation and extubation in the OR where the anesthetic was performed, and non-prone positioning. The primary endpoint was the interval from end of surgery to OR exit. Mean OR time differences with/without prolonged extubation were analyzed pairwise by surgeon. The variance among surgeons was estimated using the DerSimonian-Laird method with Knapp-Hartung adjustment for the sample sizes of surgeons. Proportions were analyzed after arcsine transformation, and the inverse taken to report results.

**Results:**

There were prolonged extubations for 23% (41,768/182,374) of cases. Prolonged extubations had a mean 13.3 min longer time from the end of surgery to OR exit (95% confidence interval 12.8–13.7 min, *P* < 0.0001). That result was among the 71 surgeons each with ≥ 9 cases having prolonged extubation times and ≥ 9 cases with typical extubation times. Results were similar using a threshold of ≥ 3 cases, comprising 257 surgeons (13.2 min, *P* < 0.0001). Among the 71 surgeons with at least nine prolonged extubations, on most days with a prolonged extubation during at least one of their cases, there were > 8 h of cases and turnover times in the OR (77%, 73%-81%, *P* < 0.0001). Results were similar when analyzed for the 249 surgeons each with ≥ 3 cases with prolonged extubation (76%, *P* < 0.0001).

**Conclusions:**

Matching earlier findings, prolonged tracheal extubation times are important economically, increasing OR time by 13 min and usually performed in ORs with lists of cases of sufficient duration to treat the extra time as a variable cost.

**Supplementary Information:**

The online version contains supplementary material available at 10.1186/s12871-024-02862-6.

## Background

A prolonged time to tracheal extubation is defined as an interval from the end of surgery to extubation 15 min or longer [1]. A previous study showed that pairwise by surgeon, prolonged times to tracheal extubation were associated with an average of 12.4 min longer from the end of surgery to the patient’s operating room exit [2]. Another study showed that such prolonged times to extubation were usually (57.0%) observed in operating room days with > 8 h of cases and turnovers [3], such that the incremental time attributable to prolonged extubation can be treated as a variable cost [4, 5]. The cost is variable, rather than fixed, because typically, in hospitals with shifts ≥ 8 h (e.g., some 8-h and some 12-h), each extra minute results in an additional expense when considered from a long-term perspective [4–6]. Studies of prevalence of workload > 8 h matter because managerial epidemiology studies show that multiple operating rooms at many facilities’ have ≤ 8 h of daily workload [7–10].

The goal of the current study was to address limitations related to the generalizability of the two earlier studies. The hospital studied earlier was chosen partly because it had few pediatric surgery cases (mean patient age = 55 years, < 5% age < 18 years) [3]. The hospital’s large ambulatory surgery center was not included, thereby limiting the study (deliberately) to an inpatient surgical suite. Finally, patients undergoing surgery in the prone position were included [3]. However, different anesthetic drugs affect patient awakening and initial recovery times [1, 11, 12], not patient positioning. Rapid awakening for prone patients may be forestalled deliberately until patients return to the supine position. In the current study, we repeated the earlier studies using more years of data, while including both adult and pediatric patients and inpatient and ambulatory surgery, and while excluding patients in the prone position. Our specific goals were to obtain more accurate estimates than the original 12.4 min per prolonged extubation, and 57.0% of those extubations in rooms with > 8 h of workload. We performed the calculations as a meta-analysis of surgeons, thereby letting us also quantify the heterogeneity among surgeons’ practices, workflows, and workloads.

## Methods

The University of Iowa Institutional Review Board determined that this retrospective cohort study #202301239 does not meet the regulatory definition of human subjects research.

The time when the anesthesia provider extubated the patient was recorded using an extubation event button in the hospital's electronic health record (Epic Systems, Verona, WI) from Sunday, 9 October 2011, forwards. With the current study started in July 2023, there were 76 eight-week periods through Saturday, 3 June 2023. We studied every case at the University of Iowa (*N* = 182,374) that included general anesthesia, tracheal intubation in the operating room where the anesthetic was performed, tracheal extubation between 5 min before the end of surgery and the time of operating room exit, and absence of prone positioning. The University of Iowa is a large teaching hospital. The cases involved 574 distinct surgeons and 696 distinct anesthesia providers (Table 1) [13, 14].

**Table 1:** Characteristics of the 182,374 cases of the 574 distinct surgeons, 13,933 combinations of surgeon and eight-week period, and 23,958 combinations of surgeon, eight-week period, and binary of prolonged time to tracheal extubation or not

Characteristic	N cases	% cases
Prolonged time to tracheal extubation (≥ 15 min)	41,768	23%
Age < 18 years	26,711	15%
Received care by surgeon with < 19 cases overall	5,962	3%
Received care by pediatric surgeon, > 50% of cases’ patients’ ages < 18 years	26,711	15%
Received care by surgeon with > 50% of cases among patients having ambulatory surgery	17,777	10%
Received care by cancer surgeon, > 50% of cases of major therapeutic procedures were with cancer diagnosis	10,657	6%
Time from end of surgery to operating room recorded and therefore included in those analyses, the weighted mean (standard deviation) of 14.8 (7.5) minutes	181,750	99.7%
Turnover between cases of same surgeon on same day,^a^ the weighted mean (standard deviation) of 39.2 (11.6) minutes	65,340	36%
Operating rooms with > 8 h of cases and turnovers during regular workday	110,865	61%
Operating rooms with ≤ 8 h of cases and turnovers during regular workday	57,194	31%
Performed weekends, holidays, nights, or missing data of an operating room exit time for any case that day	14,315	8%
Anesthetic finished by trainee: resident physician, fellow, or student registered nurse anesthetist, an endpoint associated with prolonged extubations [13, 14]	84,129	46%
≥ 11 American Society of Anesthesiologists’ base units, an endpoint associated with prolonged extubations	17,958	10%
Operating room time ≥ 4 h, an endpoint associated with prolonged extubations	41,695	23%
Any neuromuscular blocking agent used	151,061	83%
Rocuronium or vecuronium administered	93,645	51%
Sugammadex administered	31,107	17%
Neostigmine administered	62,538	34%
BIS monitor used	4,026	2%
Nitrous oxide used	46,954	26%
Sevoflurane, isoflurane, or desflurane ≥ 0.2% minimum alveolar concentration 15 min before end of surgery	166,009	91%
Sevoflurane, isoflurane, or desflurane ≥ 0.4% minimum alveolar concentration 15 min before end of surgery	153,748	84%

^a^Among the “turnovers between cases of same surgeon on same day,” 31% of the turnovers occurred after a prolonged time to extubation, where 31% = 13,140/41,768, the 41,768 in the first row. The incidence was 37% when the case did not have prolonged time to extubation, where 37% = 52,200/140,606, where 52,200 = 65,340 – 13,140 and 140,606 = 182,374 – 41,768. There were significantly fewer turnovers of the same surgeons among cases with prolonged time to extubation, estimated relative risk 0.85, *P* < 0.0001

### Explanations of the independent variable and the three dependent variables

The binary independent variable’s threshold of 15-min is not arbitrary, and dichotomization does not lose economic information [15]. Anesthesiologists rated extubation times longer than 15 min as representing poor recovery from anesthesia [16]. That threshold was associated with immediate reintubation, respiratory treatments in the post-anesthesia care unit, and treatment with flumazenil and naloxone [17]. By 15 min after the end of surgery, non-anesthesia practitioners reliably were idle in the operating room waiting for extubation [18]. Briefer times to extubation generally were not associated with longer operating room times, because non-anesthesia practitioners have their own activities to be completed before the end of the case [18]. That is, a small delay in extubating during the interval while other personnel are performing activities that need to be completed before the patient can leave the operating room (i.e., parallel processing) do not result in an exit delay [18]. Unlike briefer extubation times, all (98/98) surgeons’ mean extubation times were exceeded by 15 min [2]. Finally, prolonged extubation times are largely preventable (see the first full section of the Discussion, below).

The first of the dependent variables was the interval in minutes from end of surgery to operating room exit. There was no censoring. Every patient included was intubated and extubated in the operating room and either had prolonged extubation or not. End of surgery was considered as the time recorded when the dressing was applied or, when not applicable or missing (e.g., rigid bronchoscopy, incomplete documentation), when the end of surgery was noted.

The total hours of cases, including the turnover times, in the operating room is the “workload,” as relevant to cost accounting. Turnover times are intervals from operating room exit to operating room entrance of the next case on the same day. For purposes of quantifying the room’s workload, cases starting between 6:30 AM and 7:30 PM on regular workdays were included. The second of the dependent variables was the proportion of prolonged extubations on regular workdays in operating rooms with workloads > 8 h. Turnover times longer than 120 min were counted as equaling 120 min because such extremely long turnovers are typically caused by a case cancellation or situation where the next case was not originally planned to be performed in the performed operating room (e.g., an add-on or case moved from another operating room). The third of the dependent variables was the turnover time among cases with the next case performed by the same surgeon on the same day and in the same operating room [1].

### Statistical analyses by eight-week period and surgeon categories

Details of the statistical analyses are provided in Supplemental Table 1. These follow the order in the Results section. Patients’ dependent variables such as the daily workload are not statistically independent within or among surgeons because surgical cases are scheduled partly based on expectations of time in the operating room and the operating room’s workload [19–23]. That is, lack of independence occurs because the attributes of one patient (e.g., long duration surgical procedure) influences the attributes of the surgeons’ subsequent patients on the same day and subsequent days within the period of their “block” schedule. The University of Iowa used a four-week master surgical schedule, influencing surgeons’ operating days. Therefore, batches of eight-week periods were created, an integer multiple of the master schedule’s cycle [19–23]. The eight-week periods represent, statistically, a repeated measures analysis by surgeon. Statistical analyses were performed using Stata v18.0 (StataCorp, College Station, TX).

Our study of “surgeons” does not literally mean we evaluate surgeons, but rather the multiple behavioral decisions that are made in how patients get assigned to surgeons in clinics, how operating room cases are scheduled, and how trainees or experienced nurse anesthetists are assigned to surgeons [24, 25]. Similarly, intraoperative workflow at the end of surgery differs among surgeons because they perform different categories of procedures (e.g., head and neck versus gynecological laparoscopy) [2]. These factors matter for each of the three dependent variables.

Because the statistical analyses were performed by surgeon, all covariates in the model were surgeon characteristics. Surgeons were categorized broadly based on their patients’ ages and lengths of stay, provided the surgeon had at least 19 cases total, summed among all eight-week periods. Surgeons with most (> 50%) of their patients’ ages < 18 years were considered “pediatric surgeons.” Among the remaining surgeons, those with most of their patients admitted on the day of surgery and discharged before 11:59 PM of the day of surgery were considered surgeons with most of their practice being ambulatory surgery. Among the remaining surgeons, those with most of their patients undergoing a major therapeutic procedure [26] with an oncology diagnosis, based on their procedure’s International Classifications of Diseases, Ninth or Tenth Revision Clinical Modification diagnosis codes [27, 28], were considered surgeons with most of their practice being inpatient oncology surgery.

### Statistical methods

The first of the dependent variables was the interval in minutes from end of surgery to operating room exit. For each combination of the surgeon and eight-week period, the difference was calculated between (i) the mean operating room times after surgery ended among the cases with prolonged extubation and (ii) the mean among cases without prolonged extubation. Then, for each surgeon, the mean and the standard error of the mean were calculated over the eight-week periods during which they operated. (Standard errors, not standard deviations, are reported because weights in meta-analyses are calculated from the inverse of squares of the standard errors.) Thus, each surgeon had a point estimate of the mean difference and a corresponding standard error. The mean differences were then pooled among surgeons to obtain the final estimate, using a random intercept model. The variance of the model was estimated using the DerSimonian-Laird method [29, 30]. Knapp-Hartung adjustment was made for the sample size of surgeons [31, 32]. This random-effects analysis was performed primarily among the combinations of surgeons and eight-week periods with at least nine cases with prolonged extubation and at least nine that were not. The criterion for statistical significance was that the two-sided *P* < 0.05. Similarly, 95% two-sided confidence intervals were calculated. Sensitivity analyses were calculated using three subgroups of surgeons (pediatric surgeons, surgeons with primarily ambulatory surgery cases, and cancer surgeons) and using two different numbers of observations per eight-week period (≥ 3 and ≥ 19). For the sensitivity analyses, to adjust for the five comparisons, *P* < 0.01 was treated as statistically significant, and 99% confidence intervals were used.

The second of the dependent variables was the proportion of prolonged extubations in rooms with > 8 h of cases and turnover times. Because the observed percentage was 57% in the earlier study [3], a value significantly greater than 50% was expected but vastly less than 90%. Arcsine transformation was made of the observed proportion for each combination of the surgeon and eight-week period. [13, 19, 23, 33–35],^1^ The mean and standard error of the mean were calculated over the eight-week periods for each surgeon. Then, random intercept meta-analysis was used to estimate the overall mean and confidence interval among surgeons. The inverse transform was then reported.

The third of the dependent variables was turnover times. Analyses were planned to be like the first of the three dependent variables (i.e., interval from end of surgery to room exit). However, we knew that the sample size would be much smaller because an earlier study at the hospital [36], and studies of all hospitals in the states of Iowa and Florida [37–40], showed that most surgeons’ cases are not followed by another case of the same surgeon that day.

We had a priori criteria for the appropriateness of the sample size for the first and second of the three dependent variables. In the earlier papers from 2013, a mean difference in the time from the end of surgery to room exit exceeding 5 min was considered economically important [2]. That is not to say that briefer times would be unimportant, but rather that hospitals reliably would try to save an average of 5 min with teams working late. Therefore, we would consider our sample size sufficient if the resulting confidence interval for the mean difference excluded plus and minus 5 min (e.g., the lower limit exceeded 5 min) [2]. We expected that criterion to be satisfied because, in the 2013 paper, the standard error of the mean was 0.6 min although there were many fewer cases (72,051 versus the current study’s 182,374) and surgeons (98 versus the current study’s 574) [2]. The minimum of 5 min matters economically when the workload is large, ≥ 8 h. At most (> 50%) surgical facilities nationwide, most operating rooms have ≤ 8 h of cases and turnover times [7–10]. We tested the fractions based on 50%, that most of the prolonged extubations occurred in operating rooms with > 8 h of cases and turnovers [3]. We expected the sample size sufficient to test “most” because our previous studies had much smaller sample sizes and yet reliably detected significant difference [3].

Statistical analyses were repeated using mixed effects models to evaluate if estimates were similar. The random effect was the surgeon. Prolonged extubation was treated as a fixed effect. The eight-week periods were entered as a centered, continuous variable (i.e., the overall mean by case within each period was subtracted from each value during that period). Robust variance estimation was used. These results were secondary for two reasons. First, the mixed effects model assumes homogeneity of the effect of prolonged extubation among surgeons, known, a priori, to be false. Second, the mixed effects model treats the dependent variables of the cases of the same surgeon to be statistically independent, although that is not true for cases within periods because of staff scheduling and assignment [13]. For example, for the second dependent variable of the duration of the workday, the sample size of the mixed effect logistic regression model was the number of cases. Thus, three cases on the same day in the same operating room with > 8 h of cases and turnovers would contribute *N* = 3 although the cases encompass one scheduled workday.

## Results

### Minutes from the end of surgery to the patient’s operating room exit

**Fig. 1:**
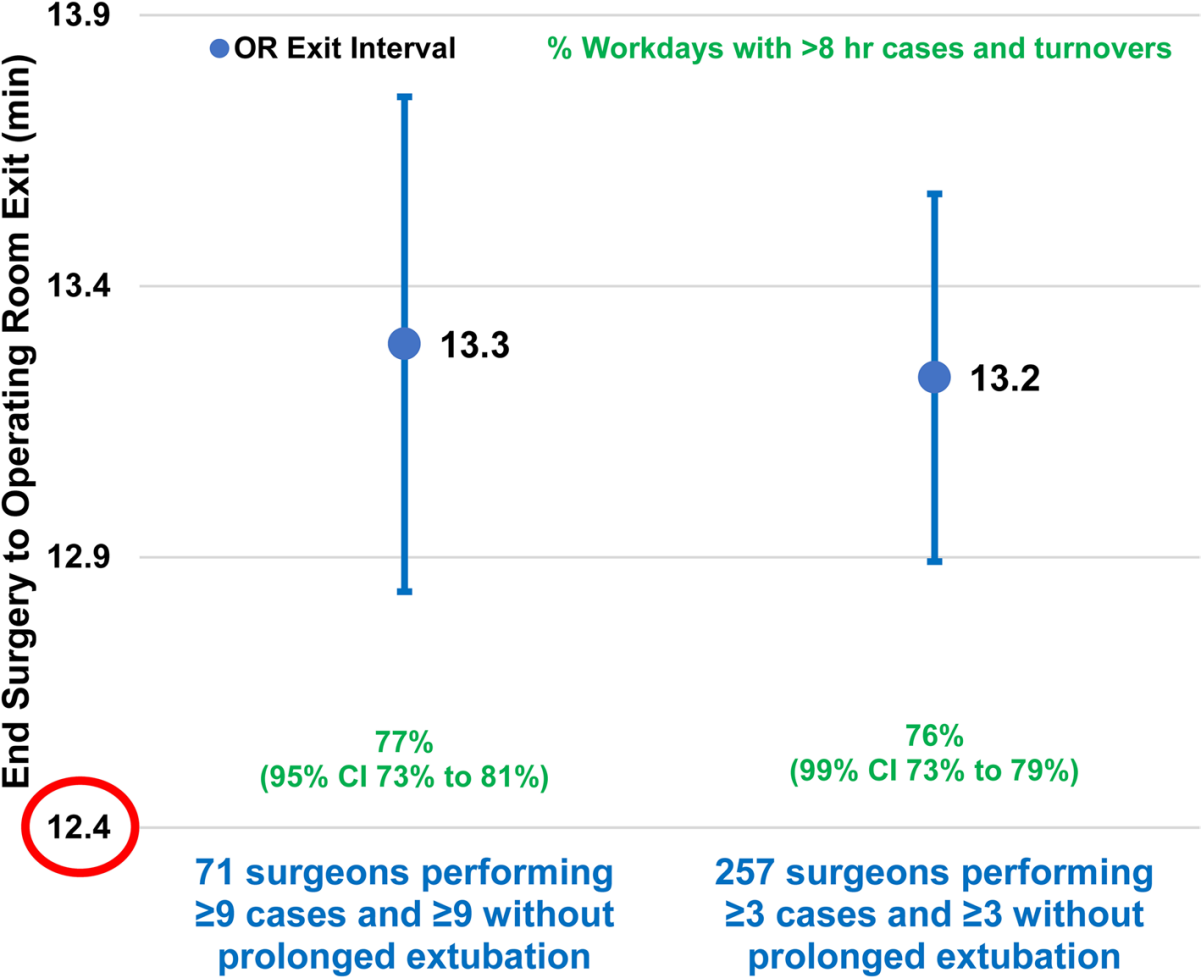
Graphical portrayal of the influence of prolonged times to tracheal extubation on the interval from the end of surgery to operating room exit. The green percentages show the frequency when the increase in time would represent a variable cost from a long-term perspective, including (when rational) small revisions to staff scheduling [4, 5]. The threshold used was 8 h because, for suites with workloads less than 8 h, small differences in operating room time are not independently associated with significant increases in overutilized time [4, 5]. The sample sizes of 71 and 257 surgeons, respectively, shown in the figure, had at least one eight-week period with least 9 (or 3) cases with a prolonged extubation and at least 9 (or 3) without a prolonged extubation. The sample sizes for the percentages shown in green font were 71 and 249 surgeons, respectively, with at least one eight-week period of at least 9 (or 3) cases with a prolonged extubation during a regular workday. There is the value of 12.4 min with red circle because our previous study estimated that, pairwise by surgeon, prolonged extubations were associated with mean 12.4 min longer from end of surgery to operating room exit (standard error of the mean 0.6 min) [2]. The objective of the current study was to address the multiple limitations, with the earlier study, listed in the Introduction

Pairwise by the surgeon, cases with a prolonged time to tracheal extubation had mean 13.3 min longer times from end of surgery to operating room exit compared to cases without prolonged extubations (95% confidence interval 12.8 to 13.7 min, *P* < 0.0001, Fig. 1). That result was for the 71 surgeons each with at least nine cases having prolonged extubation and at least nine cases without during at least one eight-week period.^2^ Results were not significantly different between the 17/71 pediatric surgeons and the 54/71 other surgeons (mean difference 1.2 min [99% confidence interval −0.2 to 2.6 min], *P* = 0.024). Results were not significantly different between the 7/54 surgeons with most of their practice ambulatory surgery and the 47/54 otherwise (*P* = 0.34).^3^ Results also were not significantly different between the 5/47 surgeons with most of their practice oncology and the 42/47 otherwise (*P* = 0.24). Results were similar when analyzed for the 257 surgeons each with at least three cases having prolonged extubation and at least three cases without (mean difference 13.2 min, 99% confidence interval 12.9 to 13.6 min, *P* < 0.0001). Results were similar when analyzed for the four surgeons each with at least nineteen cases having prolonged extubation and at least nineteen cases without (12.5 min, 99% confidence interval 10.7 to 14.3 min, *P* < 0.0001). Finally, the results were similar when analyzed for all 573 surgeons using mixed-effects modeling (13.8 min, 99% confidence interval 13.5 to 14.2 min, *P* < 0.0001, Fig. 1).

### Workload of operating rooms with prolonged extubation

When surgeons’ patients had prolonged times to tracheal extubation, on most days there were > 8 h of cases and turnover times in their operating rooms (77%, 95% confidence interval 73% to 81%, *P* < 0.0001, Fig. 1). That result was for the 71 surgeons with at least nine cases having prolonged extubation during regular workdays of an eight-week period. Results differed significantly between the 17/71 pediatric surgeons and the 54/71 other surgeons (*P* = 0.0007). The pediatric surgeons’ prolonged extubations were in operating rooms with a workload > 8 h for 67% of workdays (99% confidence interval 58% to 76%, *P* = 0.0001). In contrast, the 54/71 surgeons caring principally for adults had prolonged extubations with workloads > 8 h for 80% of workdays (99% confidence interval 75% to 84%, *P* < 0.0001). Results were not significantly different between the 9/54 surgeons with most of their practice ambulatory surgery and the 45/54 otherwise (*P* = 0.97). Results were not significantly different between the 5/45 surgeons with most of their practice oncology and the 40/45 otherwise (*P* = 0.64). Results were similar when analyzed for the 249 surgeons each with at least three cases having prolonged extubation during regular workdays of at least one eight-week period (76% of workdays, 99% confidence interval 73% to 79%, *P* < 0.0001, Fig. 1). Results were similar when analyzed for the four surgeons each with at least nineteen cases having prolonged extubation (79%, 99% confidence interval 30% to 99%, *P* = 0.038). Finally, we used mixed effects logistic regression with the data for all 455 surgeons with at least one prolonged extubation. The estimate for the fraction of prolonged extubations that were in operating rooms with workloads ≥ 8 h was 66% (99% confidence interval 64% to 68%, *P* < 0.0001; see footnote a).

### Minutes from patient’s exit from the room until the surgeon’s next patient’s entrance

Prolonged times to tracheal extubation were not significantly associated with turnover times between successive cases of the same surgeon (mean difference −0.8 min, 95% confidence interval −6.3 to 4.6 min, *P* = 0.43). That result was for 4 surgeons, the number with ≥ 9 turnovers after prolonged extubation and ≥ 9 turnovers after non-prolonged extubation, during at least one eight-week period (Table 1). Results were similar when analyzed for the 118 surgeons each with ≥ 3 turnovers after prolonged extubation and ≥ 3 turnovers after non-prolonged extubation (mean difference 0.1 min, 99% confidence interval −0.8 to 1.0 min, *P* = 0.80). When analyzed using the data for all 422 surgeons with at least one turnover time, the mean difference using mixed-effects modeling was 0.9 min (99% confidence interval 0.3 to 1.5 min, *P* < 0.0001).

## Discussion

One of our goals was to obtain a more accurate estimate for the increase in operating room time from prolonged times to tracheal extubation. We obtained 13.3 min, not differing significantly for pediatric surgeons or those performing primarily ambulatory surgery. Another goal was to confirm that substantially more than half the prolonged extubations occur in operating rooms with workdays ≥ 8 h. Because ≈77%, the increased operating room time resulting from prolonged extubations reliably can be treated as a variable cost rather than a fixed cost [4, 5]. These results match earlier ones [2, 3]. Being a variable cost, similar hospitals’ incremental costs ≈ its cost per minute of operating room time × 13.3 min per prolonged extubation (e.g., $20.47 in California × 13.3 = $272.30) [3, 4, 41].

Reevaluating the economics was important as results differed for the third endpoint (hypothesis) [1]. Previously, it was found for a different hospital that prolonged extubation times were associated with longer time from operating room exit to procedure start of the surgeon’s next case in the same operating room on the same day [1]. The mean was approximately 4.9 min (*P*< 0.0001 compared to 0). The current results seem inconsistent in that there was no (mean difference 0 min) difference in time from one patient’s operating room exit to the entrance of the next patient in the same room, when analyzed by surgeon. However, the current studied hospital was missing many incision times, and thus the same comparison could not be made. That is one potential explanation for the discrepancy. Our examination of this third study endpoint seemed to us reasonable, nonetheless, because prolonged extubations are associated with a greater workload in the post-anesthesia care unit [17]. Another potential contributor to the apparent discrepancy of findings was that the earlier study was an analysis by case, not surgeon, consistent with the current study’s finding by case of a small but statistically significant 1 min prolongation of turnover time.

### Prolonged times to tracheal extubations are modifiable

Causes of prolonged extubations are well understood, recently reviewed [15], modifiable [42, 43], and not the topic of the current article. Incidences of prolonged extubations can be reduced markedly (e.g., desflurane has ≈65% fewer prolonged extubations relative to sevoflurane and ≈78% fewer relative to isoflurane) [1, 11, 44]. Among paired patients undergoing long duration (≥ 4 h) procedures, while one cohort of patients receiving remifentanil and desflurane had prolonged extubations for 6% of cases, those with none receiving those drugs had an incidence of 39% [45]. Among the 42% of patients with end-tidal inhalational agent concentration at the end of surgery < 0.4 of the age‑adjusted minimum alveolar concentration (MAC fraction), 12% (5402/43703) had prolonged extubations, while the 18% with MAC fraction 0.4 to 0.6 had 22% (4134/18750) prolonged extubations and the other 40% of patients with MAC fraction > 0.6 had 37% (15,087/41220) prolonged extubations [43]. Individual anesthesiologists, nurse anesthetists, and resident physicians do not influence prolonged extubations substantively once controlling for their non-random assignments to cases (e.g., why we used batches of eight-week periods for statistical analyses) [46]. Rather, prolonged extubations occur more often when the anesthesia provider caring for the patient at the end of surgery has not previously finished at least five cases with the surgeon within the past three years [13, 24, 25, 43], such assignment decisions being associated with the surgeon and thus addressed appropriately in the current study. Conceptually, some patient conditions would seem likely to be significantly associated with more prolonged extubations (e.g., patients found to have difficult airways upon tracheal intubation) [47]. They are (thankfully) uncommon and so drop out of statistical models for prolonged extubations in lieu of the above-mentioned characteristics: surgical procedure (e.g., case duration) [15, 43, 46], anesthetic drugs [1, 11, 15, 44], anesthetic doses (MAC fractions) [42, 43], and staff assignments [13, 15, 24, 25, 43].

### Motivation for studying subgroups of surgeons

We performed the current study for subgroups of surgeons relevant to comparisons of sugammadex and neostigmine: surgeons principally with pediatric cases, ambulatory surgery cases, and oncology cases. Our overall and subgroup results are important for efforts to measure the impact of implementing anesthesia societies’ guidelines for reversal of neuromuscular blockade. Earlier systematic reviews with meta-analyses showed that sugammadex compared to neostigmine reduced the mean (*P* = 0.032) [48] and standardized mean (*P* = 0.007) [49] times to tracheal extubation. We updated the meta-analyses through July 1, 2023, and calculated the relative risk of prolonged times to tracheal extubation from the reported means and standard deviations of extubation times [50, 51]. The estimated relative risk was 0.514 (95% confidence interval 0.28 to 0.96, *P* = 0.038, *N* = 18 studies, Supplemental Tables 2–9). Therefore, our retrospective cohort study shows that prolonged times to tracheal extubation should be included as a cost in economic analyses of neuromuscular reversal.

### Prolonged times to tracheal extubation do not increase post-anesthesia care unit costs

Post-anesthesia care unit (PACU) costs were not included in the current study because they are not increased by prolonged extubations (i.e., their absence from the current study is not a limitation). Prolonged extubations are associated with longer post-anesthesia care unit times, mean 2.27 h (standard deviation 1.00 h) versus 2.05 h (0.93 h), *P*= 0.0022 [45]. However, even much larger increases in PACU time are associated with no greater hospital costs [52–54]. This is for two reasons [54]. First, PACU time generally is a fixed cost from the perspective of adverse events [54–56], because PACU staffing rationally is planned to a high percentage of the expected total workload to avoid the greater cost of the operating room waiting for PACU entry [57–60]. Second, even if PACU time were a variable cost, the marginal increase in cost from the prolonged extubation would not be the value of the extra PACU time, but the time multiplied by the difference in labor cost between the PACU and surgical ward [54, 59]. Using 2018 US dollars, that would be $1.85 per case with prolonged extubation [54, 59].

### Limitations, and the strength of many surgeons studied

Our study was limited to data from a single hospital. However, there was considerable heterogeneity among surgeons (I^2^ = 83% for mean differences in operating room times and I^2^ = 88% for proportions of workloads > 8 h); see Supplemental Table 1. Such large heterogeneity was fully expected, and its presence increased our confidence in the generalizability of results. The considerable heterogeneity of effect among surgeons also addresses why the mixed-effects model was included solely to confirm that the conclusions do not differ between the models. While the mixed effects model has random baseline differences among surgeons in time from the end of surgery to extubation, the model treated the incremental effect of prolonged extubation as homogeneous among surgeons, although the effect was markedly heterogeneous. That is why the mixed effects model quantitative results differ significantly from those of the other models but give insight because the implications are the same.

Although the results were limited to being from one hospital, one strength was that the primary endpoint of time from end of surgery to operating room exit had a mean difference between groups (95% confidence interval 12.8 to 13.7 min) similar to that from a different hospital (mean 12.4 min, standard error 0.6 min) [2], despite differences in patient characteristics. In addition, we do not appreciate how errors in recording the precise time of the end of surgery would differ between groups, given that prolonged extubation criteria occurs 15 min later. Nevertheless, another study strength is that the current hospital’s anesthesia information system (Epic) differed from the Innovian (Dräger, Telford, PA) system of the previously studied hospital [61].

## Conclusions

Prolonged times to tracheal extubation increase operating room time by approximately 13 min and, when they occur, frequently are in operating rooms sufficiently full for the time to be treated as a variable cost. At hospitals with many operating room days with workdays exceeding 8 h, the prevention of prolonged extubations has substantial potential to reduce costs, regardless of whether the surgeon cares principally for pediatric patients, performs inpatient or ambulatory surgery, or cares for inpatient oncology patients.

## Supplementary Information

Supplementary Material 1.

## Data Availability

All Stata computer code and statistical output are in Supplemental Table 1. The corresponding Stata data file is available after data use agreement with the University of Iowa.

## Abbreviations

OR: Operating room
PACU: Post-anesthesia care unit

## Footnotes

1 Consider three hypothetical surgeons. A was present for 1 eight-week period and had 684 prolonged extubations. B was present for 9 eight-week periods, each with 76 prolonged extubations. C was present for 76 eight-week periods, each with 9 prolonged extubations. Our analysis with arcsine transformation gives the three surgeons weights of 1, 9, and 76, because the cases and dates of prolonged extubations may be correlated (e.g., due to staff assignment of anesthesia providers [13]). Eight-week periods are long enough to be statistically independent [35].

2 There were a few (0.5% [40/7635]) surgeon periods with ≥9 cases having prolonged times to tracheal extubation but < 9 cases without prolonged extubation. As stated in the Methods, all Stata meta-analysis outputs are given in Supplemental Table 1.

3 The 7/54 may seem small, because based on patients undergoing general anesthesia with tracheal intubation and among surgeons with at least nine prolonged extubations.
